# Activation of p38 MAP kinase and stress signalling in fibroblasts from the progeroid Rothmund–Thomson syndrome

**DOI:** 10.1007/s11357-012-9476-9

**Published:** 2012-09-22

**Authors:** Terence Davis, Hannah S. E. Tivey, Amy J. C. Brook, Julia W. Grimstead, Michal J. Rokicki, David Kipling

**Affiliations:** Institute of Cancer and Genetics, School of Medicine, Cardiff University, Heath Park, Cardiff, CF14 4XN UK

**Keywords:** Telomerase, Progeroid syndrome, Cellular senescence, Tumour suppressor p53, Ageing, Stress-associated MAP kinases

## Abstract

Rothmund–Thomson fibroblasts had replicative lifespans and growth rates within the range for normal fibroblasts; however, they show elevated levels of the stress-associated p38 MAP kinase, suggestive of stress during growth. Treatment with the p38 MAP kinase inhibitor SB203580 increased both lifespan and growth rate, as did reduction of oxidative stress using low oxygen in some strains. At replicative senescence p53, p21^WAF1^ and p16^INK4A^ levels were elevated, and abrogation of p53 using shRNA knockdown allowed the cells to bypass senescence. Ectopic expression of human telomerase allowed Rothmund–Thomson fibroblasts to bypass senescence. However, activated p38 was still present, and continuous growth for some telomerised clones required either a reduction in oxidative stress or SB203580 treatment. Overall, the evidence suggests that replicative senescence in Rothmund–Thomson cells resembles normal senescence in that it is telomere driven and p53 dependent. However, the lack of RECQL4 leads to enhanced levels of stress during cell growth that may lead to moderate levels of stress-induced premature senescence. As replicative senescence is believed to underlie human ageing, a moderate level of stress-induced premature senescence and p38 activity may play a role in the relatively mild ageing phenotype seen in Rothmund–Thomson.

**Electronic supplementary material:**

The online version of this article (doi:10.1007/s11357-012-9476-9) contains supplementary material, which is available to authorized users.

## Introduction

Rothmund–Thomson syndrome (RTS) is a rare autosomal recessive genetic disorder (Thomson 1936; Rothmund 1868). The most characteristic feature is cutaneous poikiloderma (a skin condition that consists of areas of increased and decreased pigmentation, prominent blood vessels or telangiectasias, and thinning of the skin) that usually appears in infancy, with red oedematous plaques and blistering occurring on the cheeks, forehead and ears, and is most severe in sun-exposed areas. Other features include short stature, hair greying (although rarely), alopecia, sparse eyebrows and eyelashes, café-au-lait spots and hyperkeratotic lesions. It can be further categorised into two clinical subtypes: type I, which is distinguished by poikiloderma and juvenile cataracts, and type II, which is characterised by poikiloderma, congenital bone defects and osteosarcoma (Larizza et al. 2010). However, a major distinction is the presence or absence of *RECQL4* mutations (see below). Many of the clinical features of RTS are associated with normal ageing, and for this reason, it is classified as a premature ageing syndrome even though RTS individuals are felt to have a normal lifespan in the absence of cancer (Larizza et al. 2010; Jin et al. 2008; Hofer et al. 2005). Despite having been first described in 1868, only 300 or so individuals with RTS have been described in the literature, and relatively, little is known about the syndrome.

*RECQL4*, the gene mutated in RTS Type II, encodes a 1208 amino acid protein that shares significant homology with the RECQ helicases WRN and BLM that are mutated in Werner and Bloom syndromes, respectively (Liu 2010). It is found in both the cytoplasm and nucleus, and has two nuclear localisation signal domains located in the N-terminal part of the protein (Yin et al. 2004; Burks et al. 2007). RECQL4 is a member of the superfamily II (SFII) helicase family (Kitao et al. 1998). It possesses 3′–5′ helicase activity in vitro and is capable of unwinding DNA structures that include splayed arms, bubbles and blunt-end duplex DNA (Xu and Liu 2009).

Most mutations in *RECQL4* are frame-shift, nonsense or splicing mutations that are predicted to result in a truncated protein (Larizza et al. 2010; Wang et al. 2003; Bachrati and Hickson 2003; Kitao et al. 1998). It is thought that complete loss of RECQL4 is lethal in humans as complete absence of RECQL4 is incompatible with cell viability (Abe et al. 2011). In addition, ectopic expression of recombinant RECQL4 proteins containing only the N-terminal 496 amino acids in RECQL4 null cells is sufficient for cell viability (Abe et al. 2011), and the majority of *RECQL4* mutations are found 3′ to amino acid 496 (Larizza et al. 2010). These data suggest that the mutant proteins found in RTS individuals are partially active even though they may be expressed at very low levels (Ouyang et al. 2008). Interestingly, mutations in *RECQL4* have only been found in type II RTS, and the aetiology of type I RTS is unknown (Larizza et al. 2010).

Several attempts have been made to generate mouse RTS models, with mixed success. Mutations in the N-terminal domain of mouse *Recql4* are embryonic lethal (Ichikawa et al. 2002), and deletions in the SFII helicase domain proved to have high mortality, with most pups not surviving beyond 2 weeks (Hoki et al. 2003). Those animals that did survive had skin atrophy, hair graying, hair loss, short stature, bone dysplasia, dystrophic teeth and immunological abnormalities, although they failed to develop poikiloderma, bilateral cataracts or malignancies, all of which are hallmark features observed in RTS individuals (Hoki et al. 2003). The lack of these hallmark features may not be surprising, however, as the very short lifespan of these mice may leave insufficient time for their development. These mice also did not have inflammation. Finally, a *Recql4* mouse model was generated by introducing a frame-shift resulting in Recql4 truncated in the first half of the SFII helicase domain that mimics the mutation seen in many RTS individuals (Mann et al. 2005). This mouse showed hyper-pigmented skin and developed poikiloderma reminiscent of that seen in RTS individuals, defects in skeletal development and cancer pre-disposition. The *Recql4* mice that survived into adulthood had normal lifespans (Mann et al. 2005).

Features of premature ageing may be found in RTS individuals and is seen in the RTS mouse model. Replicative cellular senescence (Hayflick and Moorhead 1961) has been postulated as a factor underlying human ageing (Faragher et al. 2009; Ostler et al. 2002), and several observations suggest that senescent cells occur in vivo and accumulate with age [reviewed in (Faragher et al. 2009)]. Other data that provide support for a role of replicative cellular senescence in human ageing comes from studies of progeroid syndromes such as Werner (WS), Hutchinson Gilford Progeria Syndrome (HGPS) and Ataxia Telangiectasia (AT) [reviewed in (Davis et al. 2009)]. In these syndromes, fibroblasts show accelerated ageing in vitro, leading to speculation that this may underlie some of the accelerated features of ageing in these syndromes.

In particular, young (low population doublings) WS fibroblasts have very short lifespans and growth rates, together with an altered morphology reminiscent of senescent cells, even though they are a growing population (Davis et al. 2005a). They also show high levels of stress-associated p38 MAP kinase activity, and it has been demonstrated that continuous treatment with the p38 inhibitor SB203580 normalises their replicative lifespan and aged morphology (Davis et al. 2005a).

The MAP kinase p38 responds to and is activated by endogenous and exogenous cellular stress (Freund et al. 2011). p38 is involved in growth arrest in response to the expression of oncogenes such as *ras* (Wang et al. 2002; Deng et al. 2004), exogenous stress such as arsenite treatment or oxidative stress (Guay et al. 1997), and in telomere-dependent senescence (Iwasa et al. 2003): indeed, p38 defines a common senescence signalling pathway (Iwasa et al. 2003). p38 is important for senescence growth arrest due to its ability to activate both the p53/p21^WAF1^ and pRb/p16^INK4A^ growth arrest pathways.

These data, together with the effects of p38 inhibition on WS fibroblast growth, suggest that activation of p38 may be responsible for many of the senescence-like features of WS fibroblasts, a situation known as stress-induced premature senescence (SIPS) (Davis and Kipling 2006; Toussaint et al. 2000) and may underlie the accelerated ageing seen in WS individuals. In addition, because WS individuals have an increased prevalence of inflammatory diseases such as atherosclerosis and have high circulatory levels of p38-inducible pro-inflammatory cytokines such as TNFα (Yokote et al. 2004), it is possible that p38 activity may also underlie the inflammatory features seen in WS (Davis and Kipling 2006).

Although WS is a segmental progeria and many RTS individuals have been described as showing premature ageing, it is unknown whether they share an underlying causal mechanism. However, because RTS shares many clinical features with WS, it is possible that premature cellular ageing and p38 activation may also underlie some of the features seen in RTS. A possible inducer of p38 activation is genomic instability, and consistent with this hypothesis is the observation that karyotype analysis of RTS primary fibroblasts and lymphocytes reveals an unusually high incidence of chromosomal rearrangements, translocations and deletions (Der Kaloustian et al. 1990; Lindor et al. 1996; Orstavik et al. 1994). In addition, some RTS fibroblasts show increased sensitivity to ionising radiation (Smith and Paterson 1982; Kerr et al. 1996; Varughese et al. 1992) and UV radiation (Vasseur et al. 1999; Shinya et al. 1993). Together with a requirement for RECQL4 in DNA replication (Hoki et al. 2003; Mann et al. 2005) and telomere maintenance (Ghosh et al. 2012), these observations suggest that loss of RECQL4 may adversely impact upon DNA replication and repair, and in turn affect cellular growth and lifespan. However, few studies have been undertaken to date on the growth characteristics of RTS fibroblasts.

To address the impact of loss of human RECQL4 function on cell behaviour, we have therefore analysed multiple aspects of the RTS cellular phenotype, with particular reference to those relevant to replicative senescence and stress signalling, using primary fibroblasts from RTS individuals. We compare these data (including growth rates, replicative lifespan, the role of p53 and telomerase in determining replicative lifespan, and the effects of alleviating oxidative stress) with our previous published data on primary WS fibroblasts and discuss how the differences in cellular phenotype that we observe may explain key difference in the clinical presentation of these two progeroid syndromes.

## Methods

### Cells and cell culture

Primary dermal fibroblasts derived from biopsies of human tissue from three RTS individuals (AG18371, AG18375 and AG17524) and one normal 12-year-old individual (AG16409) were obtained from the Coriell Cell Repository (Camden, NJ). Of these, AG18371 obtained from a 12-year old is homozygous for a *RECQL4* gene deletion (g.2746del11); AG17524 obtained from a 4-year old has a compound heterozygote for a substitution (g.2626G>A) resulting in alternative splicing and a 2-bp deletion (g.4644delAT) that in turn causes a frame-shift; and AG18375 obtained from a 22-year old has a compound heterozygote for two *RECQL4* mutations (g.2626G>A and g.2886delT). All the above alleles result in protein-truncation 3′ to the nuclear localisation signal. To avoid confusion when referring to cell strains, a suffix has been added to the strain code, i.e. AG18371(RTS) for *R*othmund–*T*homson *S*yndrome or AG16409(N) for *N*ormal cells.

Cells were grown in Earle’s modified Eagle medium (EMEM) [1× MEM, 1 mM sodium pyruvate, 1× vitamins, 1× essential amino acids (all from Gibco, Life Technologies, Paisley, UK), 0.22 % (*w*/*v*) NaHCO_3_, 2 mM glutamine (both from Invitrogen, Life Technologies, Paisley, UK), 1× non-essential amino acids, 10,000 U/ml penicillin, 10 mg/ml streptomycin (all from Sigma, Poole, UK), 10 % (*v*/*v*) FCS (Autogen Bioclear, Salisbury, UK)] at 37 °C in an atmosphere containing 20 % O_2_ and 5 % CO_2_ with the medium changed daily as previously described (Davis et al. 2005a). For growth experiments using low oxygen, the O_2_ tension was reduced to 3 %. Population doublings (PDs) were calculated according to the formula: ${\mathrm{PD}} = \log \left( {{N_{\mathrm{t}}}/{N_{\mathrm{o}}}} \right)/\log 2$, where *N*_t_ is number of cells counted and *N*_o_ is number of cells seeded. Cells were passaged when attaining approximately 90 % confluency. Initial growth rates were calculated over the first 30 days of culture.

### Inhibitor treatments

For SB203580 treatment, cells were fed daily with EMEM supplemented with 2.5 μM SB203580 (Tocris Chemical Co., Bristol, UK), and for controls, an equivalent volume of the drug solvent (DMSO) was added to the medium; 2.5 μM is within the range used routinely for studying the effects of SB203580 on p38 activity in cell biological systems (Wang et al. 2002; Haq et al. 2002; Iwasa et al. 2003). We have previously shown that this concentration effectively inhibits p38 but does not significantly inhibit the related JNK1/2 kinases (Bagley et al. 2010).

The protein kinase A (PKA) inhibitor H89 was obtained from Tocris Chemical Co. (Bristol, UK) as the hydrogen chloride salt. H89 dissolved in DMSO was added to culture dishes to a final concentration of 10 μM (Shiryaev et al. 2011).

### BrdU incorporation assays

DNA synthesis was assayed by labelling cells in the presence of 10 μM bromodeoxyuridine (BrdU) for 1 h, following which BrdU incorporation was detected by immunoperoxidase as previously described (Davis et al. 2003). The proportion of BrdU-positive cells was assessed in a total count of 500 cells. Assays were done in triplicate.

### Detection of senescence-associated β-galactosidase activity

Endogenous mammalian senescence-associated β-galactosidase activity (SAβ-gal) was assessed histochemically as described (Davis et al. 2003).

### Retroviral gene transfer

For ectopic expression of telomerase pBABE-hTERT, an amphotropic retrovirus expressing the catalytic protein subunit of human telomerase hTERT was constructed by cloning the *Eco*RI insert of pGRN121 (a gift from Geron Corp, Menlo Park, CA, USA) into pBABE-puro (Morgenstern and Land 1990) and packaged in ψCRIP cells. For control infections, pBABE-puro vectors packaged in ψCRIP cells were used.

A shRNA targeted to p53 was expressed from pMKO.1PS (kindly donated by William Hahn, Harvard Medical School) packaged in ψCRIP (Moffat et al. 2006). The p53 21-bp short hairpin sequence is 5′-GACTCCAGTGGTAATCTACTG-3′. Empty pMKO.1PS packaged in ψCRIP cells was used as the puromycin control.

Retroviral transduction was carried out as described previously (Davis et al. 2003). Fibroblasts for retroviral infection were plated in 6-cm Petri dishes 24 h prior to infection at a seeding density of 6,000 cells/cm^2^. Prior to infection, cells were treated with polybrene at 8 μg/ml for 1 h. Cells were infected by treating with 4 ml filtered retroviral supernatants for 24 h, before replacing the supernatant with 5 ml of fresh EMEM without selection. Forty-eight hours after the start of infection, fibroblast cultures were passed into EMEM containing puromycin (2.5 μg/ml), at serial dilutions of 1:5, 1:10, 1:100 and 1:250, and observed for colony development. Alternatively, fibroblasts were passaged into fresh dishes with selective medium and cultured as described above to produce bulk cultures of mixed clones.

### Detection of telomerase activity

Telomerase activity in whole cell extracts was detected using the telomeric repeat amplification protocol (TRAP) assay as described previously (Davis et al. 2005b). The cell line 293 provided a telomerase-positive control. Reaction products were separated on non-denaturing 10 % polyacrylamide gels and visualized by Sybr Gold staining and fluorimaging on a STORM system using blue fluorescence mode (AP Biotech).

### Immunoblot analysis

Protein samples were prepared in lysis buffer containing the phosphatase inhibitors NaF and Na_3_VO_4_, separated on 12 % sodium dodecylsulphate/polyacrylamide electrophoresis gels, electroblotted to Immobilon-P polyvinylidene difluoride membrane (Millipore, Watford, UK) and antibodies applied as described previously (Davis et al. 2005a, b). The antibodies used were: mouse monoclonal anti-p53 (DO-1) (Calbiochem, Merck, UK); mouse monoclonal anti-p21^WAF1^ (6B6), rabbit anti-p16^INK4^ (both from Becton Dickinson, Oxford, UK); mouse monoclonal anti-HSP27 (G31), rabbit polyclonal anti-phospho(Ser82)-HSP27, anti-p38, anti-phospho(Thr180/Tyr182)-p38, anti-MK2 (all from Cell Signalling, New England Biolabs, Hitchin, UK); and rabbit polyclonal anti-actin (Sigma, Poole, UK). An enhanced chemiluminescence kit (GE Healthcare, Amersham, UK) was used for visualization using HRP-coupled goat secondary antibodies.

### Immunofluorescence microscopy

Actin staining for immunofluorescence microscopy was performed as described (Davis et al. 2005a). Briefly, the cells were plated into 35-mm plastic dishes in EMEM and allowed to settle for 48 h. The cells were then washed in phosphate-buffered saline (PBS), fixed in 3.7 % paraformaldehyde for 20 min and permeabilised with 0.1 % Triton-X100 for 20 min. F-actin was detected using fluorescein isothiocyanate (FITC)-conjugated phalloidin (33 μg/ml) (Sigma, Poole, UK), diluted 1:50 in PBS for 30 min in the dark, followed by washing in PBS.

## Results

### Lifespan, growth rate and morphology of primary RTS fibroblasts

**Fig. 1:**
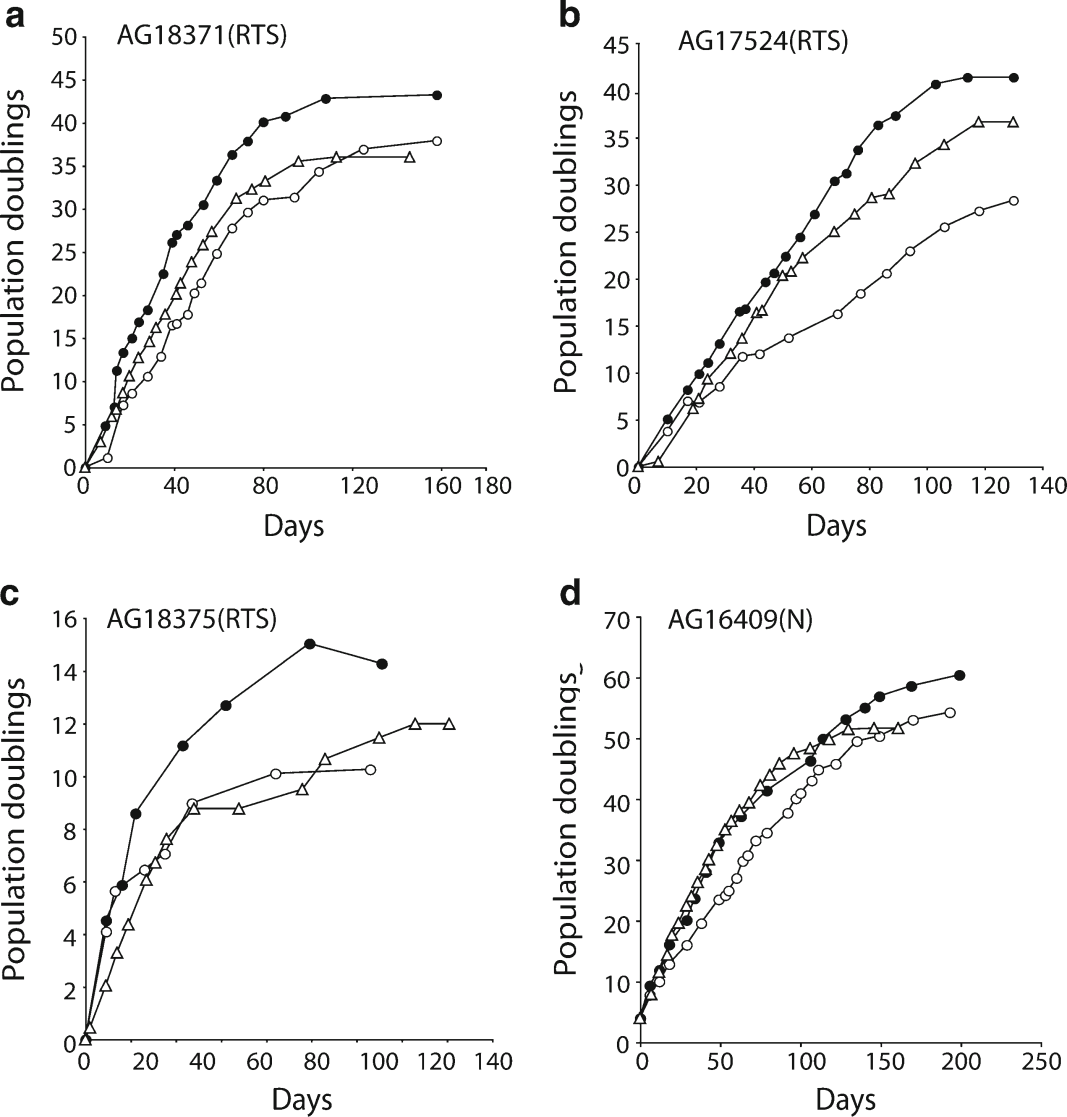
Growth of Rothmund–Thomson syndrome and normal fibroblasts. Fibroblasts were grown in standard EMEM with no supplementation (*open circle*), with continual daily supplementation with SB203580 (*solid circle*), under 3 % oxygen (*open triangle*). Growth measured as PDs versus days

Three strains of primary RTS dermal fibroblasts were grown to M1 senescence, and the effects of SB203580 or culture under low oxygen were determined and compared to normal fibroblasts grown in parallel (Fig. 1). Growth rate, cellular lifespan and morphology were determined.

The normal strain of dermal fibroblasts, AG16409(N), had a replicative lifespan of 54.3 PDs that increased to 61.9 PDs with SB203580 treatment and decreased to 51.7 PDs under low oxygen (Table 1). In contrast, the replicative lifespans of all three RTS strains were less than for the control AG16409(N) fibroblasts, irrespective of treatment. The three RTS strains had a mean replicative lifespan of 25.5 ± 14.0 PDs under control conditions, and all showed an increase in lifespan when treated with the p38 inhibitor SB203580. The overall lifespan (33.0 ± 16.3 PDs) of the RTS strains in the presence of SB203580 corresponds to a lifespan extension of 33 %. Growth under reduced (3 %) oxygen conditions has a more variable effect on RTS fibroblast lifespan, and although the mean lifespan was increased to 28.3 ± 11.5 PDs (Table 1), this is largely the result of a strong response by AG17524(RTS).

**Table 1:** Lifespan and growth rates of normal and RTS fibroblasts

Strain	Growth condition^a^	Lifespan achieved (PDs)	% lifespan increase^b^	Growth rate (PDs/day)^c^
AG16409(N)	Control	54.3	n/a	0.41
	SB203580	61.9	11.4	0.65
	3 % oxygen	51.7	−4.8	0.55
AG18371(RTS)	Control	37.9	n/a	0.41
	SB203580	43.2	14.0	0.70
	3 % oxygen	36.0	−5.1	0.52
AG18375(RTS)	Control	10.3	n/a	0.24
	SB203580	14.3	38.8	0.37
	3 % oxygen	12.0	16.5	0.22
AG17524(RTS)	Control	28.3	n/a	0.31
	SB203580	41.4	46.3	0.46
	3 % oxygen	36.7	29.7	0.41

*n/a* not applicable

^a^Cells grown in EMEM (control), in EMEM + 2.5 μM SB203580 or in EMEM under 3 % oxygen

^b^The percent increase is determined by lifespan achieved in SB203580, or under 3 % oxygen, divided by control lifespan

^c^Value determined for the growth during the first 30 days

The mean initial growth rate (determined over the first 30 days of growth) for RTS cells was 0.31 ± 0.08 PDs/day. This increased to 0.51 ± 0.17 and 0.38 ± 0.17 PDs/day for SB203580 treatment and under low oxygen, respectively. The initial growth rate increased in all RTS strains under all treatments with the exception of AG18375(RTS) under low oxygen (Table 1). At early passage, RTS cells had BrdU labelling indexes (LIs) ranging from 23.1 ± 1.9 % for AG18375(RTS) to 28.5 ± 1.9 % for AG18371(RTS), versus 31.8 ± 2.1 for AG16409(N) cells. For each strain, the BrdU LI increased with SB203580 treatment or when grown under conditions of low oxygen reflecting the increased initial growth rate seen for each cell strain, with the exception of AG18375(RTS) cells grown under low oxygen (data not shown).

**Fig. 2:**
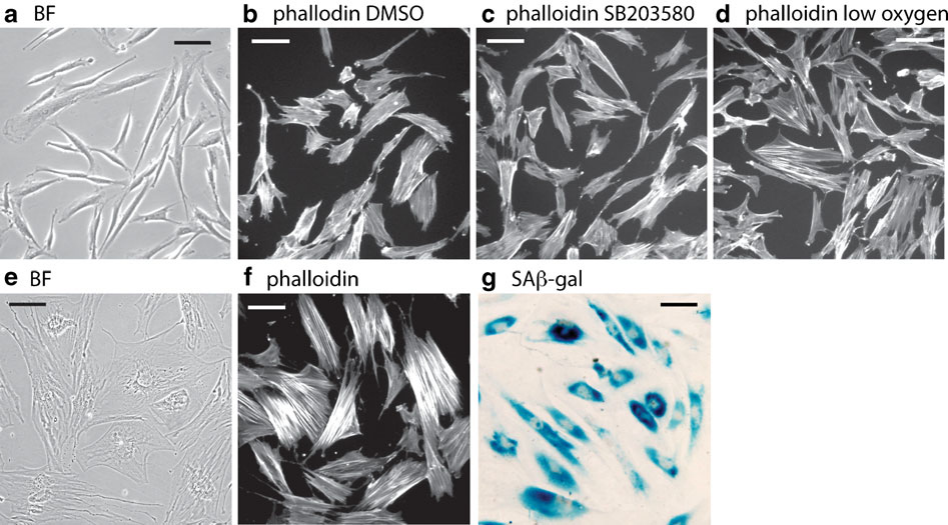
Morphological analysis of AG18371(RTS) fibroblasts. **a**–**d** Cycling cells, **e**–**g** cells at M1. BF are cells under phase contrast, phalloidin are cells stained with phalloidin-FITC, SAβ-gal are cells stained for senescence-associated β-galactosidase activity. *Bar* = 100 μm

The RTS strains showed a consistent morphology when young (i.e. at low PD), as illustrated by the AG18371(RTS) cells shown in Fig. 2a. These were mostly small in size and resembled AG16409(N) cells (Fig. S1, see Online resource 1). When stained with FITC-phalloidin most of the young AG18371(RTS) cells were small, with few F-actin stress fibres visible, and only a few cells had an enlarged morphology (Fig. 2b). SB203580 treatment or growth under conditions of reduced oxygen tension had little effect upon the morphology and stress-fibre phenotype of the AG18371(RTS) cells (Fig. 2c, d). Similar results were seen for the AG18735(RTS), AG17524(RTS) and AG16409(N) strains (Fig. S1, see Online resource 1).

At M1 senescence, the AG18371(RTS) cells had an enlarged and irregular morphology (Fig. 2e) with extensive F-actin stress fibres (Fig. 2f), very low BrdU LI (1.2 ± 0.5 % or less) and a high proportion of SAβ-gal staining (see dark patches in Fig. 2g). Similar results were seen for the AG18375(RTS) and AG16409(N) cells (Fig. S1, see Online resource 1). AG17524(RTS) cells at M1 senescence were slightly atypical, in that they had the enlarged morphology, low BrdU LI and high SAβ-gal staining as seen for the other RTS strains, but did not show extensive F-actin stress fibres (Fig. S1, see Online resource 1). Note that, although we have described here a single NDF, we have provided growth data for a further six NDF strains (Table S1, See Online resource 2).

### Abrogation of p53 function extends the cellular life span of RTS fibroblasts

To determine the role of p53 in the regulation of RTS fibroblast lifespan, pre-senescent AG18371(RTS) fibroblasts at PD 36 (BrdU LI of 3.4 ± 0.8 %) were infected with amphotropic retroviral vectors encoding a puromycin resistance (*puro*) gene alone, or both *puro* and an shRNA to p53. Analysis of puromycin-resistant colonies [designated (RTS)^puro^ and (RTS)^p53^, respectively] began 2 to 3 weeks after infection.

**Fig. 3:**
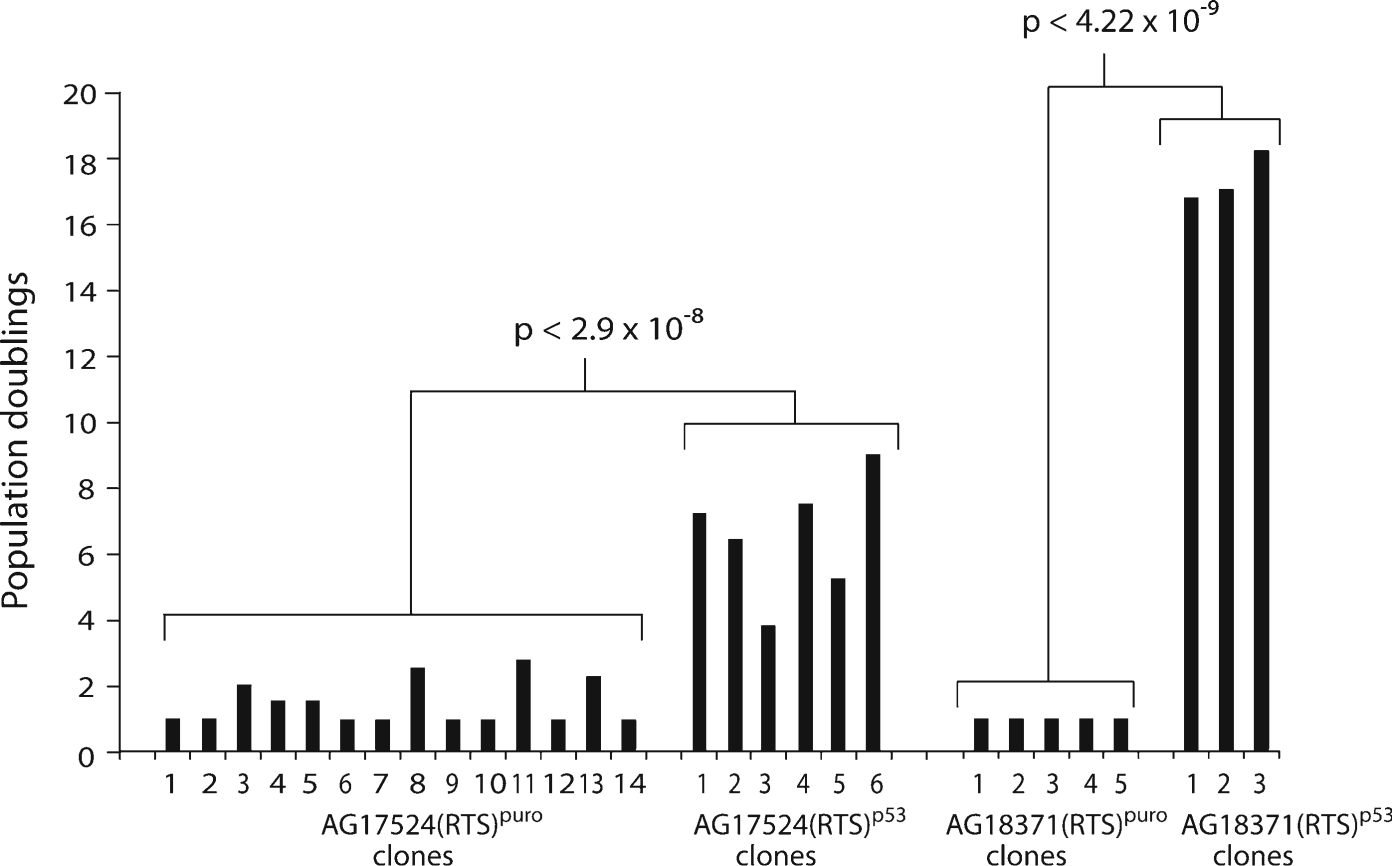
Extension of cellular lifespan of Rothmund–Thomson cells following abrogation of p53. Bar chart showing lifespan of puro and shp53 expressing clones. Statistical test is Student’s *t* test

**Fig. 4:**
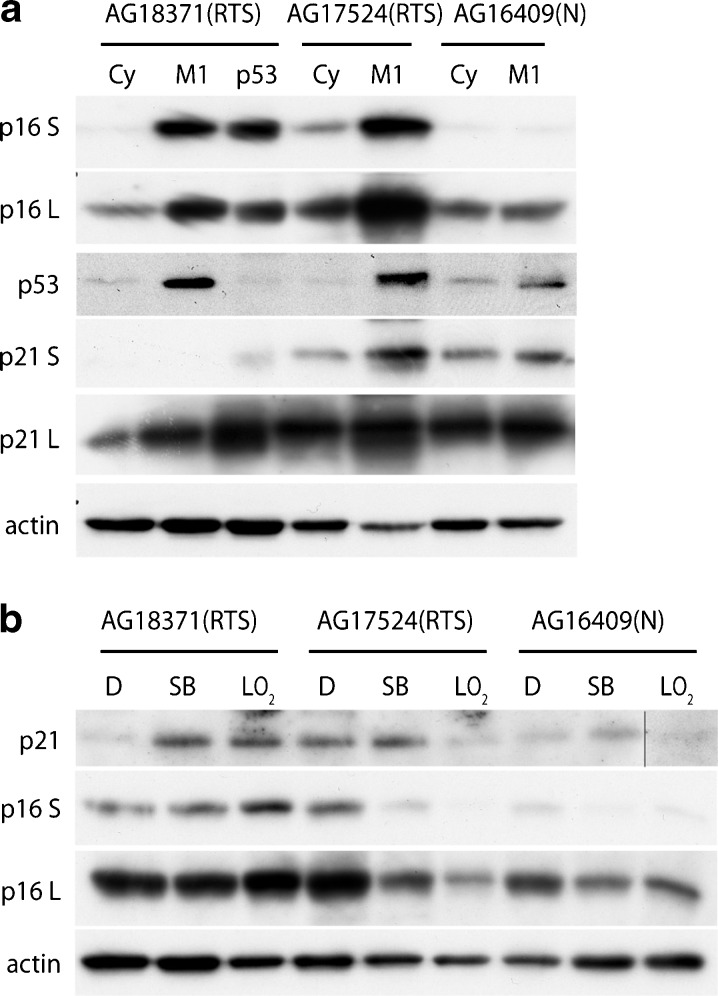
Immunoblot analysis of cell cycle proteins in RTS and normal cells. **a** Lysates were prepared from cycling cells (*Cy*), senescent cells (*M1*) and cycling shp53 cells (*p53*). **b** Lysates were prepared from cells grown in EMEM + 0.1 % (*v*/*v*) DMSO (*D*), EMEM + 2.5 μM SB203580 (*SB*), cells grown under low oxygen (*LO*_*2*_). Expression levels were compared for various proteins as indicated: *S* and *L* indicate short and long exposures. Actin was used as a loading control. Note that the p21 band for AG16409(N) LO_2_ in **b** has been cut and pasted from the same gel and exposure as the other p21 bands (indicated by vertical black line) but has otherwise been treated exactly as for the rest of the gel

Cells expressing *puro* alone ceased proliferating within the first 2 weeks and entered a senescent-like state with clones of a maximum of two cells (1 PD) (Fig. 3). The AG18371(RTS)^puro^ cells at this stage appeared essentially identical to uninfected AG18371(RTS) cells at M1 (not shown). In contrast, expression of the p53 shRNA resulted in evasion of senescence and generated rapidly growing colonies. In AG18371(RTS)^p53^ cells at about 15 PDs post-infection (13 PDs post M1), p53 protein levels were very low compared to cells at M1, showing that the shRNA had successfully abrogated p53 levels (Fig. 4a). At 15 PDs post-infection, the AG18371(RTS)^p53^ cells are approaching M^int^ (see below).

Young AG18371(RTS)^p53^ cells (<10 PD post-infection) had a small morphology (not shown) and a BrdU LI of 25.2 % ± 1.8 %. Eventually, more than 120 days post-infection, net growth ceased (Fig. 3) and the cells entered a state similar to a second senescence-like state termed M^int^ that has been described previously for p53-abrogated human fibroblasts (Bond et al. 1999). The M^int^-like state for the three AG18371(RTS)^p53^ clones was reached with a final cell count equivalent to 17.35 ± 0.77 PDs post-infection (Fig. 3); this compares to 1.0 PD for the five puro control clones (*p* < 4 × 10^−9^; Student’s *t* test). A similar but less dramatic lifespan extension was seen for AG17524(RTS) cells (Fig. 3); when infected at PD 26.5, the puromycin-resistant control clones managed an average post-infection lifespan of 1.31 ± 0.67 PDs before reaching M1, compared to 6.52 ± 1.82 PDs for shRNA-expressing AG17524(RTS)^p53^ cells at M^int^ (*p* < 2.9 × 10^−8^; Student’s *t* test). The short lifespan and thus limited cell numbers available for AG18375(RTS) precluded analysis of this strain.

### Expression of cell cycle proteins in RTS and normal fibroblasts

Molecular profiling of the normal fibroblasts and two of the RTS strains was undertaken by immunoblot analysis; limited cell numbers for AG18375(RTS) precluded analysis of this third RTS strain. Cycling RTS cells showed low levels of the cyclin-dependent kinase inhibitors (CdkIs) p21^WAF1^ and p16^INK4A^ that increased markedly when the cells reached M1 (Fig. 4a). This contrasts with the normal cells where the level of p16^INK4A^ did not increase in AG16409(N) cells at M1, whereas there was a small increase in p21^WAF1^ (Fig. 4a). The levels of the CdkIs appeared to be higher in cycling AG18371(RTS) cells than in cycling AG17524(RTS) or AG16409(N) cells; however, the CdkI levels were low in all the cycling cell populations (Fig. 4b). Treatment with SB203580 or growth at low oxygen did not affect the levels of the CdkIs in cycling AG18371(RTS) but appeared to reduce p16^INK4A^ in cycling AG17524(RTS) and AG16409(N) cells (Fig. 4b). SB203580 treatment or growth under low oxygen had only a small effect on the levels of p21^WAF1^.

p53 protein was present at low levels in cycling RTS and AG16409(N) cells. This increased in AG16409(N) at M1 senescence, and even more markedly so in RTS cells at M1 (Fig. 4a). Interestingly, despite loss of p53 expression in AG18371(RTS)^p53^ cells, the level of the p53 target p21^WAF1^ actually increased in the AG18371(RTS)^p53^ cells, presumably due to p53-independent up-regulation. In contrast, levels of p16^INK4A^ in AG18371(RTS)^p53^ cells remained the same as seen at M1. Insufficient numbers of cells were available for a similar analysis of p53 and CdkI expression in AG17524(RTS)^p53^.

### RTS fibroblasts have activated p38 signalling

**Fig. 5:**
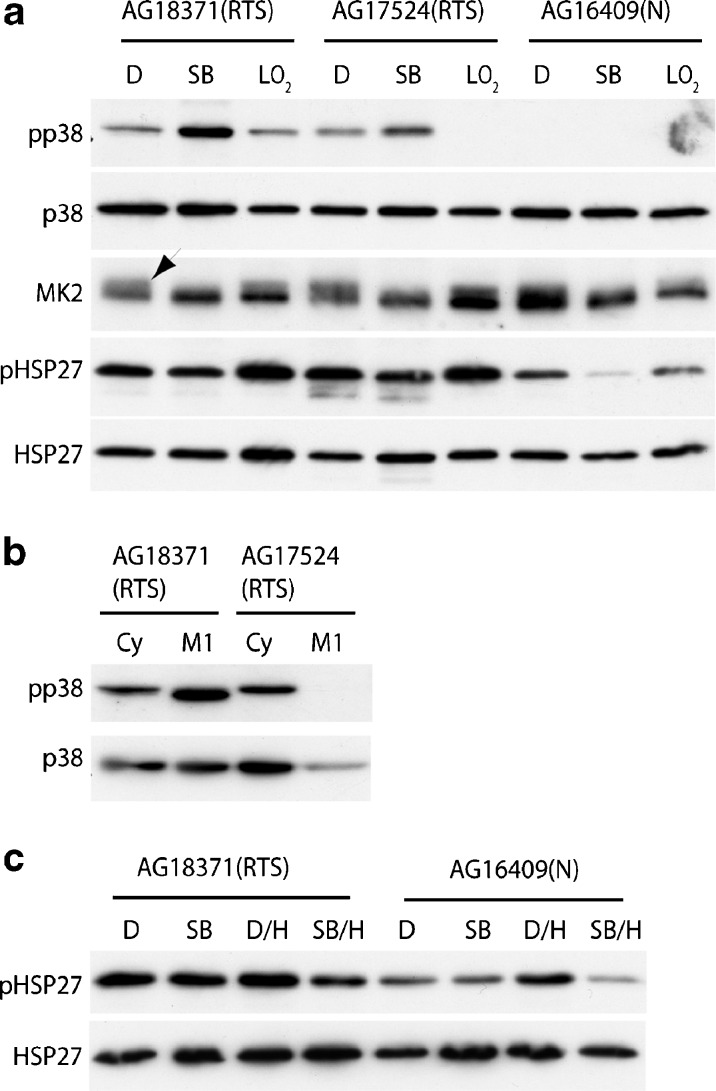
Analysis of stress kinases in RTS and normal cells. **a** Lysates were prepared from cells grown in EMEM + 0.1 % (*v*/*v*) DMSO (*D*), EMEM + 2.5 μM SB203580 (*SB*), cells grown under low oxygen (*LO*_*2*_). **b** Lysates were prepared from cycling cells (*Cy*) and senescent cells (*M1*). **c** Lysates were prepared from cells grown for 3 days in EMEM + 0.1 % (*v*/*v*) DMSO. Cells grown for 3 days in EMEM + 0.1 % (*v*/*v*) DMSO followed by 10 μM H89 for 2 h (*D/H*), cells grown for 3 days in EMEM + 2.5 μM SB203580 (*SB*), cells grown for 3 days in EMEM + 2.5 μM SB203580 followed by 10 μM H89 for 2 h (*SB/H*). Expression levels were compared for various proteins as indicated; pp38 and pHSP27 are the phosphorylated (activated) versions of p38 and HSP27, respectively. Samples were equalised for p38 expression (*a* and *b*) or HSP27 (*c*). In **a**, the *arrow* marks activated MK2

Activated (phosphorylated) p38 was detected by immunoblot assay in both AG18371(RTS) and AG17524(RTS) young primary fibroblasts but not in young AG16409(N) cells (Fig. 5a). This level of p38 phosphorylation increased further at M1 senescence in AG18371(RTS) cells but decreased in AG17524(RTS) cells at M1 even when taking into account sample under-loading (Fig. 5b). This observation correlates with the lack of stress fibres seen in AG17524(RTS) but not AG18371(RTS) cells at M1 (Fig. S1, see Online resource 1, Fig. 2f). Interestingly, p38 was activated further when young RTS cells were grown in the p38 inhibitor SB203580 (Fig. 5a).

The immediate p38 downstream target MK2 showed activation in young cycling RTS cells, as demonstrated by the presence of a doublet (Fig. 5a, arrow), and activation was also detected, albeit to a lesser extent, in AG16409(N) cells. As expected, treatment of all cells with SB203580 prevented MK2 activation, as shown by the absence of a doublet (Fig. 5a). However, growth of cells under low oxygen had little effect on MK2 activation.

HSP27, the downstream target of MK2, was phosphorylated in both AG18371(RTS) and AG17524(RTS) fibroblasts and, to a lesser extent, in AG16409(N) cells (Fig. 5a). In contrast to MK2, HSP27 phosphorylation was not reduced by SB203580 in young RTS cells, although it was in AG16409(N) cells (Fig. 5a, C), suggesting that some degree of MK2-independent phosphorylation of HSP27 is occurring specifically in RTS cells. Protein kinase A (PKA) has been implicated in F-actin rearrangements via MK5 induced HSP27 phosphorylation (Shiryaev et al. 2011). To determine whether the retention of HSP27 phosphorylation in the presence of SB203580 in RTS cells was PKA-dependent, we used the PKA inhibitor H89. Treatment of 16409(N) and AG18371(RTS) cells with H89 for 2 h had a pronounced effect upon cellular morphology (not shown) but did not alter the phosphorylation status of HSP27 (Fig. 5c). As treatment of cells with H89 for 30 min is sufficient to block PKA activity and down-regulate phosphorylated HSP27 (Shiryaev et al. 2011), this suggests that HSP27 phosphorylation in RTS cells appears not be due to either the p38 or PKA pathways.

### Extension of the lifespan of RTS cells by ectopic hTERT expression

**Fig. 6:**
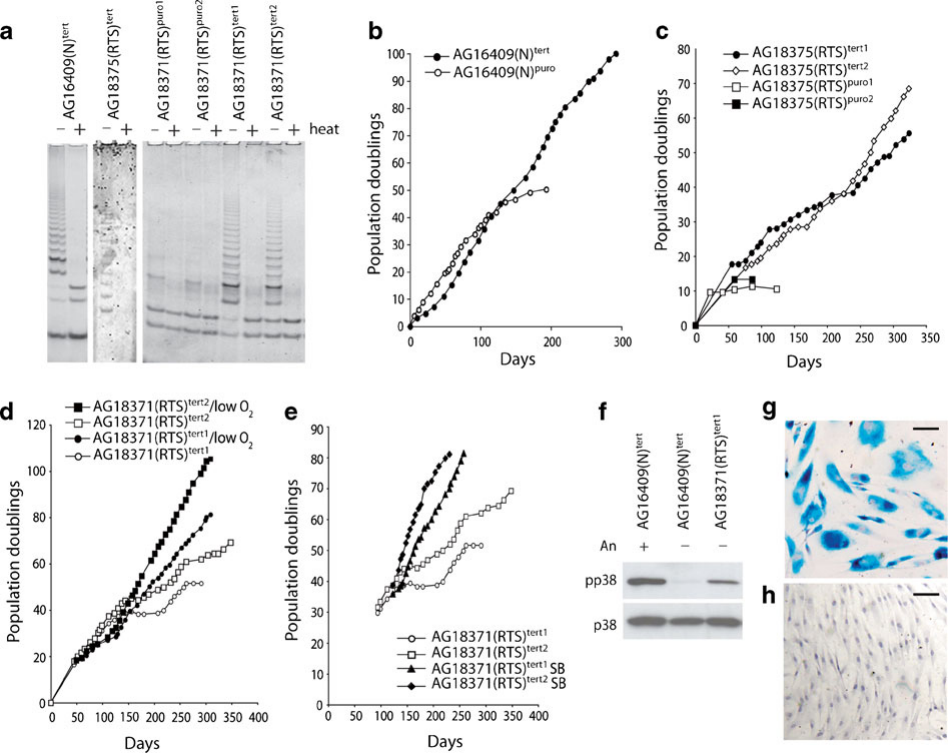
Extension of cellular lifespan of AG16409(N) and RTS cells following ectopic expression of hTERT. **a** TRAP assay showing telomerase activity: heat = samples heat-treated (+) at 85 °C for 10 min to destroy the telomerase activity. **b** Growth in PDs versus days for AG16409(N) cells. **c** Growth in PDs versus days for AG18375(RTS) cells. **d** Growth in PDs versus days for AG18371(RTS)^tert^ clones grown under 20 % or 3 % oxygen. **e** Growth in PDs versus days for AG18371(RTS)^tert^ clones grown with or without SB203580 (the *open circles* and *open squares* are the data from **d**). **f** Immunoblot showing activated p38 in AG18371(RTS)^tert1^ cells; the controls are AG16409(N)^tert^ cells and AG16409(N)^tert^ cells treated with 30 μM anisomycin (Sigma, Poole, UK) for 45 min to activate p38. **g**, **h** SAβ-gal stained AG18371(RTS)^tert1^ cells at 52 PDs grown under 20 % oxygen or at 71 PDs grown under 3 % oxygen, respectively; *bar* = 100 μm

To assess the role of telomere erosion in regulating RTS cellular lifespan, early passage RTS cells were infected with retroviruses expressing puromycin resistance and hTERT (the catalytic protein subunit of telomerase), or puromycin resistance only. Drug-resistant colonies were selected and designated as (RTS)^tert^ and (RTS)^puro^. Telomerase activity was assessed using the TRAP assay (Fig. 6a). AG16409(N) cells were used as a control, and AG16409(N)^tert^ cells reached >100 PDs, double the lifespan seen in AG16409(N)^puro^ cells (Fig. 6b), and have continued to divide. Similar observations of the immortalisation of normal fibroblasts by ectopic hTERT expression have been reported numerous times previously.

For AG18375(RTS), cells were infected at 1 PD and the two AG18375(RTS)^puro^ clones managed 10.5 and 13.3 PDs before reaching M1 (Fig. 6c). The AG18375(RTS)^tert^ clones 1 and 3 achieved greater than 55 PDs and showed little sign of senescing; indeed, these lines are still growing today. The expression of telomerase activity was confirmed using TRAP assay (Fig. 6a).

For AG18371(RTS), the three puro-only control clones achieved between 25 and 35 PDs and as expected were TRAP negative (Fig. 6a and data not shown). For AG18371(RTS)^tert^, two clones expressing telomerase were selected for long-term culture. The first clone, AG18371(RTS)^tert1^, underwent two phases of almost stationary growth and ceased dividing at 52 PDs post-infection (Fig. 6c) at which time the cells showed an enlarged morphology with high staining for SAβ-gal (see dark patches in Fig. 6g) and a BrdU LI of <5 %, suggesting that these were senescent cells. The AG18371(RTS)^tert1^ cells have activated p38 compared to AG16409(N)^tert^ cells, similar to AG18371(RTS) primary cells (Fig. 6f). The second clone, AG18371(RTS)^tert2^, managed >70 PDs and is still dividing (Fig. 6d); however, this clone also underwent two phases of almost stationary growth during this time with the cells having high SAβ-gal staining and a low BrdU LI (data not shown).

Both AG18371(RTS)^tert^ clones were transferred to low-oxygen conditions (3 %) at early passage (∼18 PDs) and, after a brief period of relatively slow growth, maintained a high growth rate and have now achieved >81 PDs for AG18371(RTS)^tert1^ and >100 PDs for AG18371(RTS)^tert2^ suggestive of immortalisation (Fig. 6d). The AG18371(RTS)^tert1^ cells under low oxygen at 71 PDs resembled young AG18371(RTS) cells having a small morphology and very low SAβ-gal staining (Fig. 6h) and a high BrdU LI. Finally, transferring AG18371(RTS) clones 1 and 2 to medium containing SB203580 at 2.5 μM just prior to the first slow growth phase resulted in rapidly growing cultures (Fig. 6e).

## Discussion

Progeroid syndromes are widely used as models for some aspects of human ageing, the best-studied being WS, AT and HGPS (Martin et al. 1999; Hofer et al. 2005; Davis et al. 2009; Navarro et al. 2006; Kipling et al. 2004). Fibroblasts from WS, AT and HGPS individuals show accelerated ageing in vitro, and this may underlie some of the accelerated features of whole body ageing in these syndromes, with WS and AT fibroblasts having very abbreviated lifespans and HGPS fibroblasts showing high levels of apoptosis (Tollefsbol and Cohen 1984; Tchirkov and Lansdorp 2003; Bridger and Kill 2004). Although RTS individuals show some features of premature ageing, RTS is less well studied and relatively little has been reported to date on the growth characteristics of RTS fibroblasts, and it is unknown whether any aspects of accelerated cell ageing occur in this syndrome that might contribute to the premature ageing of these individuals. For this reason, this study sought to examine multiple aspects of the RTS cellular growth phenotype relevant to cellular ageing, including growth rates, replicative lifespan, the role of p53 and telomerase in determining replicative lifespan, and the effects of alleviating oxidative stress.

A role for RECQL4 in DNA replication has been suggested, as the primary interacting proteins are in human chromatin, including the DNA replicating factors MCM10, MCM2-7, CDC45 and GINS (Xu et al. 2009). The RECQL4-MCM replication complex is required only during the G_1_ and S phases of the cell cycle, and RECQL4 has been shown in a *Xenopus* model to be important for the initiation of DNA synthesis via the recruitment of DNA polymerase α (Sangrithi et al. 2005; Matsuno et al. 2006). In addition, the circadian protein TIMELESS and its partner protein TIPIN, which are important for replication progression, cohesion establishment and stress response, are also found complexed with RECQL4. A role for RECQL4 in DNA replication initiation has been implicated from studies using mouse embryonic fibroblasts (MEFs) from the *Recql4* SFII helicase deficient mice; these grew more slowly than normal MEFs and had reduced DNA replication (Mann et al. 2005; Hoki et al. 2003). Together, these data suggest that RECQL4 has a unique role in the establishment of DNA replication that is not seen in other RECQ proteins (Liu 2010), although the details of this role remain unclear. Despite these prior findings, we find that primary fibroblasts from RTS individuals do not show a dramatic reduction in growth rate. We find that RTS fibroblasts had growth rates that varied between 0.24 and 0.41 PDs/day (Table 1), whilst normal fibroblasts had growth rates varying between 0.15 and 0.41 PDs/day (Table S1, see Online resource 2). This suggests that any defect in DNA replication in human RTS cells is relatively subtle and insufficient to have a major impact on overall population growth rate.

Young (low PD) RTS cells show activated stress-associated p38 MAP kinase, together with activated MK2 and phosphorylated HSP27 compared to normal fibroblasts [this work and (Davis et al. 2005a; Davis and Kipling 2009; Davis et al. 2007)], yet strongly resemble normal fibroblasts in morphology and do not have extensive F-actin stress fibres. The lack of p38 activation and stress fibres is seen both in the NDF strain from a young individual used here and NDF strains from older individuals used previously (Davis and Kipling 2009). This differs markedly from young WS cells, which although showing activated p38 and phosphorylated HSP27 furthermore show extensive stress fibres (Davis et al. 2005a). WS cells have very short replicative lifespans (Tollefsbol and Cohen 1984) and much reduced growth rates (Davis et al. 2005a; Faragher et al. 1993), and it has been argued that lack of the WRN protein leads to stress-induced premature senescence (SIPS) as a result of replicative stress (Davis and Kipling 2006). Treatment of WS fibroblasts using the p38 inhibitor SB203580 corrects these phenotypes and extends the replicative lifespan to within the range seen for normal fibroblasts (Davis et al. 2005a). Although the growth rates of RTS fibroblasts were also increased with SB203580, the degree of increase was not significantly more than is seen for normal fibroblasts when treated with SB203580 (Table S1, see Online resource 2). Taken together, our data suggest that while lack of RECQL4 results in increased levels of stress signalling (as judged by elevated phospho p38 in cycling RTS cells) in comparison to normal fibroblasts, it is nevertheless at a level that does not significantly impact on cell growth rates. In contrast, WS cells show an elevated level of stress signalling that is sufficient to impact on cell growth rates, as judged by the major effect of SB203580 on WS cell growth rates (see Table 2).

**Table 2:** Comparison between normal, RTS and WS fibroblasts

Condition	Normal	RTS	WS^a^
Lifespan	25–55 PDs normal range	10–37 PDs^b^ within normal range	15–25 PDs reduced^c^
Growth rate	0.15–0.41 PDs/day normal range	0.24–0.41 PDs/day within normal range^b^	0.06–0.14 PDs/day reduced^c^
Effect of SB20580	Increased lifespan, increased growth rate	Increases within normal range^b^	Significantly increased^c^
Cell morphology	Normal, mostly small with few stress fibres	Similar to normal	Many enlarged cells with F-actin stress fibres
Effect of SB203580 on morphology	Almost no effect	Almost no effect	Cells resemble low PD normal cells
Cells at M1	Enlarged with F-actin stress fibres	AG17524(RTS) enlarged no stress fibres, others as normal cells	As normal cells
Phospho p38 level in low PD cells	Not detected	Detectable: low in AG18371 compared to M1, low in AG17524 and not seen at M1	Detectable: as high as seen at M1

^a^Data adapted from Davis et al. (2005a)

^b^Not significantly different from normal

^c^Significantly different from normal

Oxidative stress is known to play a role in SIPS via its activation of p38 MAP kinase signalling (Huot et al. 1997). This does not appear to be the case for AG18371(RTS) cells, however, since reduced oxidative stress (growth in reduced oxygen) had little effect on the growth rates or replicative lifespan of this strain and no effect on stress kinase activation. However, reduced oxygen did have an effect on AG17524(RTS) cells, resulting in an increased cellular lifespan and a reduction in p38 activation. The differences in the RTS strains may be due to the different specific *RECQL4* mutations in each strain or the broader difference in genetic background. These data contrast with that for WS fibroblasts where low oxygen increased cellular lifespan in all strains studied, although it did not affect the aged morphology or the phosphorylation of HSP27 (Kashino et al. 2003; Davis et al. 2007).

Strain AG18371(RTS) illustrates that it is possible to have the clinical RTS phenotype together with fibroblasts that show active stress signalling that does not respond to a reduction in oxidative stress. This suggests that loss of RECQL4 function may directly lead to activation of stress signalling, with the possibility that oxidative stress may synergise with this on some genetic backgrounds. Possible causes of stress in RTS cells as a direct consequence of RECQL4 function include problems occurring during chromosome segregation and/or DNA replication; RECQL4 forms a stable complex with the ubiquitin ligases UBR1 and UBR2, which are involved in the N-end rule pathway shown to be essential for correct chromosome segregation (Rao et al. 2001; Yin et al. 2004), and has a known role in DNA replication (Xu et al. 2009). In addition, stress may result from genomic instability as manifested by a high incidence of chromosomal rearrangements, translocations and deletions in RTS cells (Orstavik et al. 1994; Lindor et al. 1996; Der Kaloustian et al. 1990).

The average replicative lifespan for three strains of RTS fibroblasts (25.5 ± 14.0 PDs) is less than the normal fibroblasts used in this study (54.3 PDs). Additional data from our previous work on an additional seven normal fibroblast strains, grown under the same conditions used in this study, show that they had a mean replicative lifespan of 38.8 ± 10.5 PDs (Table S1, see Online resource 2). Although overall in this small dataset (*n* = 3 RTS strains) RTS fibroblasts do not show a statistically significant reduction of replicative lifespan compared to normal fibroblasts, it should be noted that three out of eight of the normal fibroblast strains used previously had replicative lifespans in excess of 45 PDs, and none had lifespans less than 24 PDs (this work and Table S1, see Online resource 2). In contrast, of the RTS strains, one had a lifespan of approximately 10 PDs and none had a lifespan greater than 38 PDs. Thus, although the reduction in average lifespan of RTS fibroblasts did not reach statistical significance, nevertheless the data are consistent with the RTS strains clustering in the lower part of the range of fibroblast replicative lifespans.

RTS fibroblasts at M1 resembled senescent normal fibroblasts with regard to morphology, the demonstrated low BrdU LIs, high staining for SAβ-gal activity, and elevated levels of the CdkIs p21^WAF1^ and p16^INK4A^ compared to growing cells (Bond et al. 1999; Dulic et al. 2000). p53 levels were higher in senescent RTS cells compared to matched young cycling cells, paralleling similar observations reported previously for human IMR90 fibroblasts (Chen et al. 2004) and sheep fibroblasts (Davis et al. 2005b), and contrasts with the lack of increase in p53 protein levels reported for normal human HCA2 fibroblasts and WS fibroblasts (Davis et al. 2003; Webley et al. 2000). Overall, RTS cells at senescence strongly resemble fibroblasts from other sources, and any differences may simply reflect donor or tissue-specific effects. One curious observation is the lack of F-actin stress fibres and p38 MAP kinase activation in AG17524(RTS) cells at M1, which contrasts with the high levels of activated p38 seen in AG18371(RTS) or WS fibroblasts at M1 (Davis et al. 2005a). Elevated p38 activity and F-actin stress fibres have been found in several strains of human fibroblasts at M1 (Davis et al. 2005a; Iwasa et al. 2003), and activated p38 results in production of F-actin stress fibres (Huot et al. 1997).

The elevation of p53 and p21^WAF1^ at M1 suggests a causal role in senescence in RTS cells. Indeed, experimental abrogation of p53 using shRNA expression caused RTS fibroblasts to bypass M1. Similar to what has been reported for normal fibroblasts, abrogation of p53 function does not result in complete immortalisation of RTS cells but rather causes a finite extension of cellular lifespan and eventual entry into a downstream state of non-growth termed M^int^ (Bond et al. 1999). AG18371(RTS)^p53^ cells reach M^int^ after approximately 18 PDs, a lifespan extension similar to that seen in normal and WS cells (Davis et al. 2003; Bond et al. 1999).

However, contrary to that seen in normal or WS fibroblasts (Davis et al. 2003; Bond et al. 1999; Dulic et al. 2000), the levels of p21^WAF1^ increased in the AG18371^p53^ cells. Whilst p21^WAF1^ can be up-regulated as normal cells approach M^int^ in a p53-independent manner, its level is still low compared with normal cells at M1 (Bond et al. 1999), suggesting a different p53-independent process in AG18371(RTS)^p53^ cells. As p21^WAF1^ can be up-regulated in IMR90 cells by oxidative stress even when p53 is abrogated using siRNA (Zdanov et al. 2007), it may be that AG18371(RTS)^p53^ cells have increased stress as they bypass M1, although it is not known whether there is increased oxidative stress in AG18371(RTS)^p53^ cells. Alternatively, the known chromosomal instability seen in RTS cells (Lindor et al. 1996; Orstavik et al. 1994; Der Kaloustian et al. 1990) may result in increased stress as AG18371(RTS)^p53^ cells bypass M1 leading to up-regulation of p21^WAF1^, albeit this is speculation.

Recently, it has been reported that RECQL4 is required for telomere maintenance as, although the frequency of telomere fusion and breakage was not elevated in RTS compared to normal cells, the frequency of fragile telomeres was increased in AG18371(RTS) fibroblasts, and in HeLa and U2OS cells depleted in RECQL4 using shRNA constructs (Ghosh et al. 2012). In addition, RECQL4 localises to telomeres at S phase and has weak D-loop resolving activity, at least in vitro. These data, together with the putative role for RECQL4 in DNA replication (Liu 2010), suggest that RECQL4 is involved in telomere maintenance during DNA replication, even though the growth rates of RTS cells are within the normal range (this work). As ectopic expression of telomerase in RTS cells successfully produced telomerase activity and resulted in immortalised RTS cells lines, either RECQL4 is dispensable for telomerase activity, or the low levels of RECQL4 thought to be found in RTS cell strains (Ouyang et al. 2008) are sufficient for telomerase activity and telomere maintenance in the presence of high telomerase levels.

The ability of telomerase to extend the lifespan of RTS cells was limited and cell strain dependent. In AG18375(RTS), telomerase expression extended the lifespan well beyond the puro-only controls. For AG18371(RTS), however, telomerase expression was insufficient to immortalise some clones, or resulted in poorly growing clones, unless the cells were grown under conditions of low oxygen or in the presence of p38 inhibitors. This may be due to lack of RECQL4 resulting in increased stress when telomerase is ectopically expressed, since RECQL4 is involved in telomere maintenance (Ghosh et al. 2012). This stress (or its effect on cell lifespan) appears to be reduced under low-oxygen conditions or when the p38-signalling pathway is inhibited.

Overall, the evidence suggests that lack of RECQL4 leads to enhanced levels of stress in RTS cells compared to normal cells, possibly as a result of the chromosomal instability seen in RTS and/or the reduced DNA replication. This stress is transduced by the p38-signalling pathway and leads to SIPS. However, as RTS cells do not have major differences in replicative lifespan, growth rates or cellular morphology compared to normal fibroblasts (see Table 2 for summary), this SIPS is only high enough to have minor effects on the replicative capability of RTS cells. In contrast, in WS a high level of SIPS is supported by the much reduced growth rates, replicative lifespan and the aged morphology seen in WS fibroblasts, features that are all corrected using the p38 inhibitor SB203580 (Table 2). This lower level of stress in RTS cells is further supported by the low level of p38 activation in young AG18371(RTS) cells compared to cells at M1, contrasting with the high level of activated p38 seen in both young and senescent WS cells (Table 2). A moderate level of stress is also supported by the reduced sensitivity of RTS cells to a wide range of genotoxic agents compared to WS cells (Jin et al. 2008), and reports showing little sensitivity of RTS cells to such agents (Cabral et al. 2008; Werner et al. 2006).

That the low level of SIPS in RTS cells does not lead to major proliferative defects in fibroblasts does not preclude a role of SIPS in RTS. Indeed, the progeroid features seen in RTS are mild compared to WS (Hofer et al. 2005) and parallel the lower level of SIPS seen in RTS cells in culture. In contrast, the much reduced lifespan and high levels of SIPS seen in WS fibroblasts parallel the more severe progeroid features for WS that include the inflammatory conditions of atherosclerosis and type II diabetes (Davis and Kipling 2006). If replicative lifespan of fibroblasts in vitro does reflect on whole body ageing rates, it may be that the mild progeroid features seen in RTS result, in part, from a small stress-induced reduction in replicative lifespan that is not easily detectable due to the large variations seen in both RTS and normal fibroblasts, given the unavoidably small number of RTS strains that have been studied to date. It is of course possible that the low level of SIPS and p38 activity seen in RTS fibroblasts is due to RTS not really being a bona fide progeroid syndrome: indeed, the data in this paper may be interpreted as support for that conclusion.

One of the problems in interpreting the data in this work is that the RTS phenotpyes are somewhat subtle, possibly due to RECQL4 being an essential protein and thus the mutations being hypomorphic with residual RECQL4 activity and not nulls. In addition, the lack of isogenic lines for direct comparison leads to genetic background issues. These problems may be alleviated to some extent using RNAi knockdown of RECQL4 RNA in control fibroblasts lines. However, RNAi is not without its own problems namely that the knockdown has variable penetration, thus possibly leaving detectable levels of wild-type RECQL4 that would be difficult to compare to the low levels of mutant proteins found in RTS fibroblasts. Even so, future studies should examine phenotypes in isogenic cells to help substantiate the findings in this study and to attempt to minimize genetic background effects.

## Electronic supplementary material

Below is the link to the electronic supplementary material.

Fig. S1 Morphological analysis of AG16409(N), AG18375(RTS) and AG17524(RTS) fibroblasts. Cycling cells (*rows 1–4*), cells at M1 (*rows 5–7*). Cells were grown in standard EMEM supplemented with 0.1 % DMSO (r*ows 1, 2, 5–7*), or 2.5 μM SB203580 (*row 3*), EMEM under 3 % oxygen (*row 4*). PC are cells under phase contrast, phal are cells stained with phalloidin-FITC, SAβ-gal are cells stained for senescence-associated β-galactosidase activity. *Ba*r = 100 μm (JPEG 199 kb)

High resolution image (TIFF 31302 kb)

ESM 1 (DOC 30 kb)
