# Supplementary figures and images for: Activation of p38 MAP kinase and stress signalling in fibroblasts from the progeroid Rothmund–Thomson syndrome
Source: Age (Dordr). 2012 Sep 22;35(5):1767–83. doi: 10.1007/s11357-012-9476-9 (PMC3776094; doi:10.1007/s11357-012-9476-9)

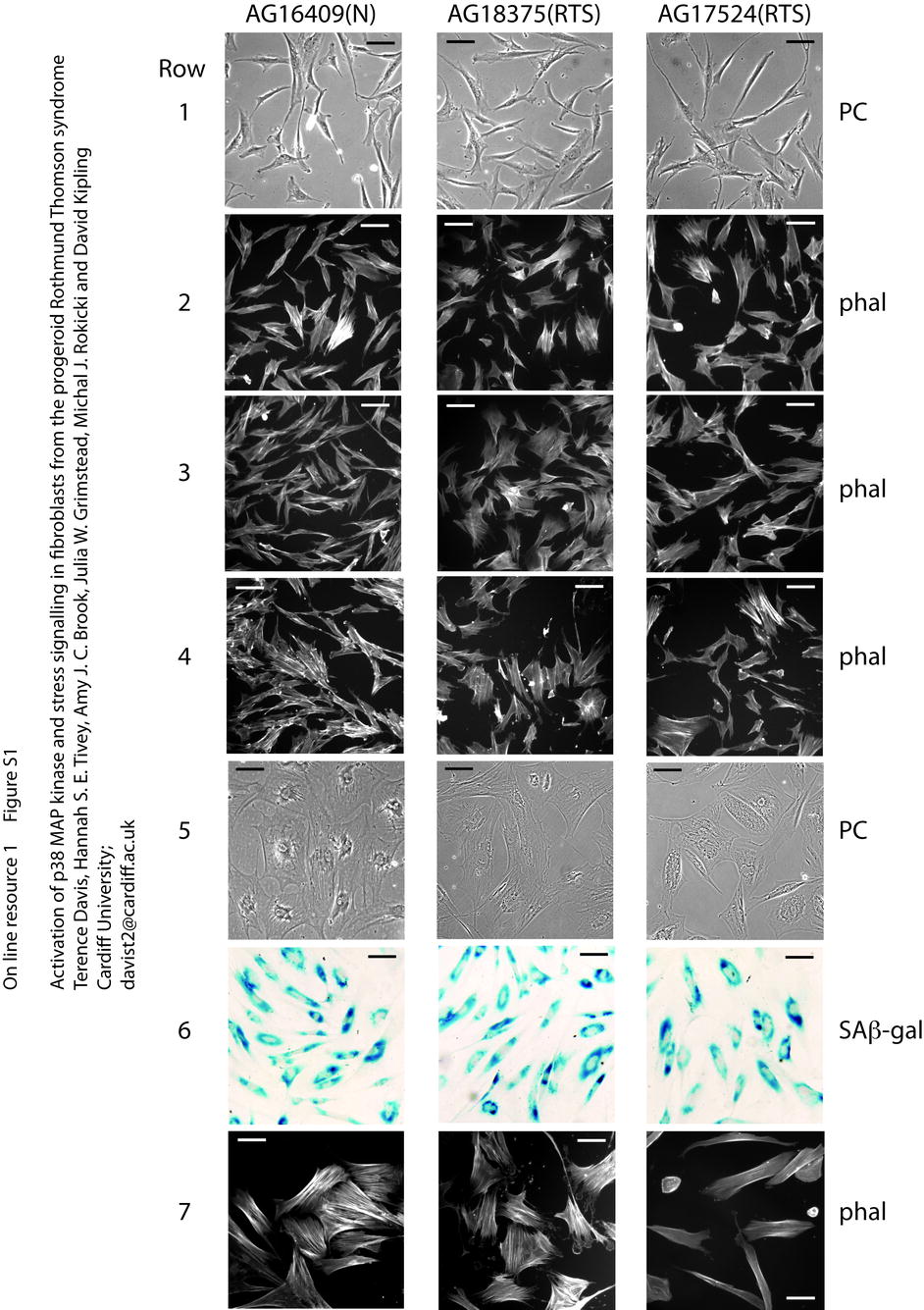

Supplement: Supplementary file 1 — Morphological analysis of AG16409(N), AG18375(RTS) and AG17524(RTS) fibroblasts. Cycling cells (rows 1–4), cells at M1 (rows 5–7). Cells were grown in standard EMEM supplemented with 0.1 % DMSO (rows 1, 2, 5–7), or 2.5 μM SB203580 (row 3), EMEM under 3 % oxygen (row 4). PC are cells under phase contrast, phal are cells stained with phalloidin-FITC, SAβ-gal are cells stained for senescence-associated β-galactosidase activity. Bar = 100 μm (JPEG 199 kb) [file 11357_2012_9476_Fig7_ESM.jpg]

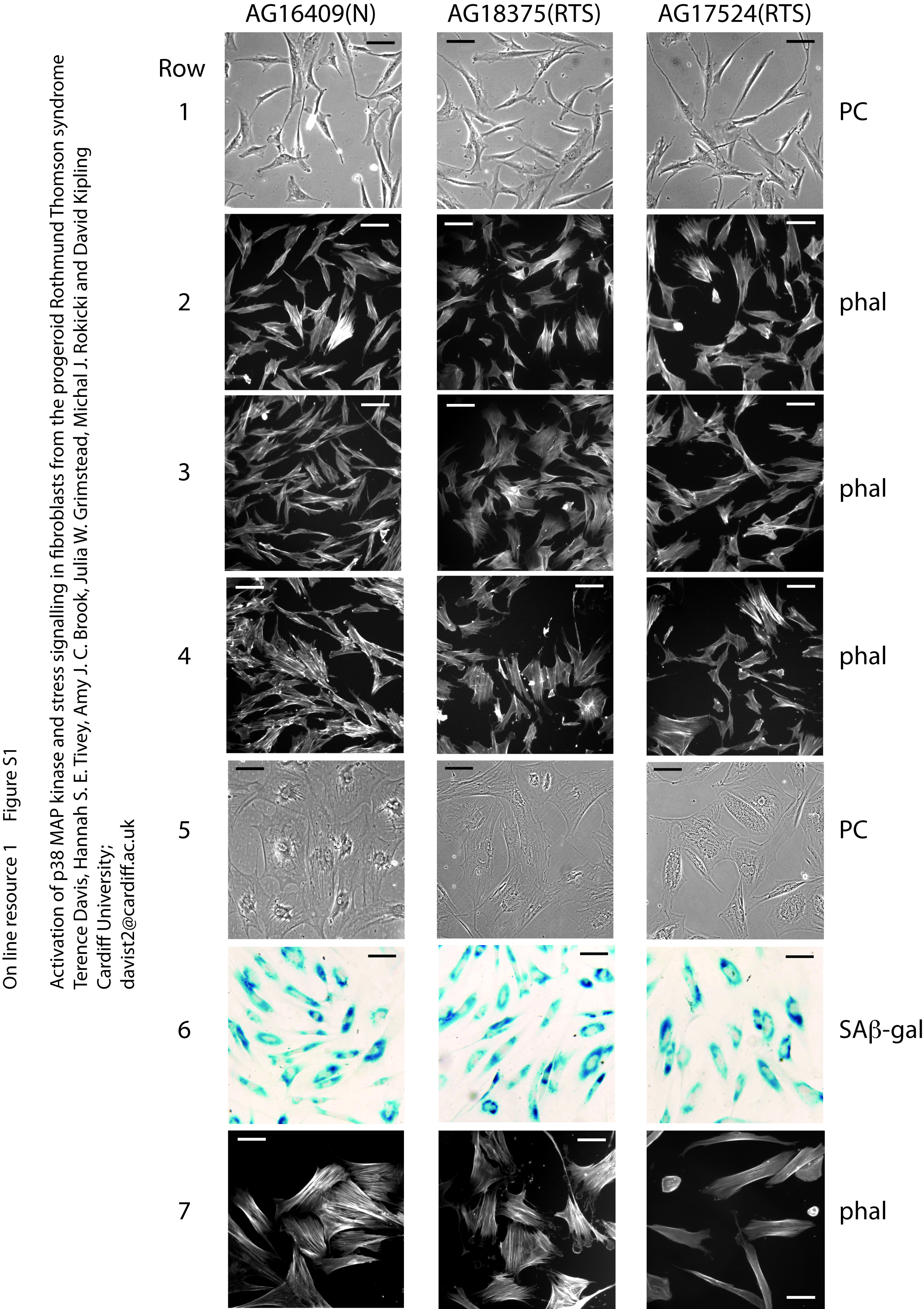

Supplement: Supplementary file 2 — High resolution image (TIFF 31302 kb) [file 11357_2012_9476_MOESM1_ESM.tif]
