# Supplementary material for: Activation of p38 MAP kinase and stress signalling in fibroblasts from the progeroid Rothmund–Thomson syndrome
Source: Age (Dordr). 2012 Sep 22;35(5):1767–83. doi: 10.1007/s11357-012-9476-9 (PMC3776094; doi:10.1007/s11357-012-9476-9)
Supplement: Supplementary file 3 — (DOC 30 kb) [file 11357_2012_9476_MOESM2_ESM.doc]

**Online resource 2**

Activation of p38 MAP kinase and stress signalling in fibroblasts from the progeroid Rothmund Thomson syndrome

Terence Davis, Hannah S. E. Tivey, Amy J. C. Brook, Julia W. Grimstead, Michal J. Rokicki and David Kipling

Cardiff University

Davist2@cardiff.ac.uk

**Table S1.** Lifespan and growth rates of normal fibroblasts

**Strain** ^a^ PDs achieved ^b^ PDs achieved ^b^ growth rate ^c^ growth rate ^c^

(control) (SB203580) (control) (SB203580)

AG04552(N) ^d^ 24.3 30.1 0.17 0.24

AG06234(N) ^e^ 34.1 41.6 0.26 0.40

AG09603(N) ^d^ 47.8 55.7 0.33 0.40

AG11020(N) ^d^ 41.7 46.5 0.39 0.45

AG11081(N) ^e^ 33.4 39.3 0.33 0.41

AG13152(N) ^d^ 28.0 35.6 0.15 0.19

AG13156(N) ^e^ 46.6 63.4 0.36 0.55

AG16409(N) ^f^ 54.3 61.9 0.41 0.65

*Mean 38.8 ± 10.5 46.6 ± 12.1 0.29 ± 0.09 0.4 ± 0.13*

^a^ All from Coriell Cell Repositories

^b^ Cells grown in EMEM (control) or in EMEM + 2.5 µM SB203580

^c^ Value determined for the growth during the first 30 days.

^d^ Data adapted from (Davis and Kipling 2009)

^e^ Unpublished data

^f^ This work
